# Hemosuccus pancreaticus as an unusual cause of upper gastrointestinal bleeding: Case report and literature review

**DOI:** 10.1016/j.ijscr.2022.107624

**Published:** 2022-09-09

**Authors:** Daniela Ayala, Juliana González T, Felipe Pedroza, Carlos Eduardo Rey Chaves, Danny Conde, Juan Carlos Sabogal Olarte

**Affiliations:** a School of Medicine, Universidad del Rosario, Bogotá D.C. 111711, Colombia; b Department of Hepatobiliary and Pancreatic Surgery, Hospital Universitario Méderi, Bogotá 111711, Colombia; c School of Medicine, Pontifical Xavierian University, Bogotá, Colombia

**Keywords:** Hemosuccus pancreaticus, Upper gastrointestinal bleeding, Angiographic embolization, Wirsungrhagia, Pseudohemobilia, Hemoductal pancreatitis

## Abstract

**Background:**

Hemosuccus pancreaticus is a rare cause of upper gastrointestinal bleeding (1/1500) and represents the loss of blood through the main pancreatic duct and its exteriorization through the major duodenal papilla. It can lead to massive bleeding, which is potentially life-threatening. This condition most commonly follows pseudoaneurysm formation due to acute or chronic pancreatitis. As a result of its infrequency, it is difficult to diagnose, and the mortality rate remains high. To our knowledge, this is the first case report on the Latin-American population.

**Case presentation:**

A 70-year-old male patient presented with diffuse severe abdominal pain associated with melaena. Angiotomography was performed, ruling out mesenteric ischemia, with evidence of pancreatic head tumor with liver metastases. Sandblom's triad was present and the diagnosis of hemosuccus pancreaticus was confirmed. Multiple arteriograms were performed, with pseudoaneurysm of gastroduodenal artery findings. Therefore, endovascular coil embolization was performed in two opportunities to control the bleeding.

**Conclusion:**

Upper gastrointestinal bleeding is a clinical challenge for the surgeon and emergency medicine. It's a complex entity with high mortality that should be suspected in patients with acute or chronic pancreatitis and periampullary tumors with non-established sources of bleeding. Clinically manifested by Sandblom's triad. Its diagnosis gold standard is arteriography plus embolization which is also therapeutic. Surgery is related to higher mortality and reserved for specific situations.

## Highlights

• Hemossucus pancreaticus is a rare condition with increased risk of mortality
• Endovascular management reduce morbidity rates
• Pancreatic bleeding should be suspected in the presence of elevated amylase, abdominal pain and gastrointestinal bleeding.

## 1 Introduction

Hemosuccus pancreaticus (HP) is a rare cause of upper gastrointestinal bleeding caused by the rupture of a pseudoaneurysm of a peripancreatic blood vessel into the pancreatic duct that leads to the duodenum [1]. Usually, local inflammatory processes lead to its development and rupture, most commonly, in the context of acute or chronic pancreatitis and pancreatic tumors, however, it has also been associated with cystic neoplasms, or iatrogenic traumatic causes [2]. It may result in obscure bleeding and massive hemorrhage that can be life-threatening [1], [2]. Diagnosis can be difficult for physicians due to its anatomical location and that bleeding into the duodenum is intermittent and cannot be easily diagnosed by endoscopy [2].

Hemosuccus pancreaticus has been estimated to occur in about one in 1500 cases of gastrointestinal bleeding [1], [3], [4]. There are no specific statistics about the morbidity and mortality associated with the uncommon presentation. [4] According to Yu et al., the mortality rate could reach up to 9.6 % [2], [5]. It is more frequent in males (7:1), with an average age between 50 and 60 years [4].

Patients with hemosuccus pancreaticus usually present with abdominal pain (65 %), gastrointestinal hemorrhage (43.5 %), and high amylase levels (40 %) [1], [3], signs and symptoms that compose the classic triad proposed by Sandblom in 1977 [6]. Abdominal pain is explained by increased intraductal pressure, caused by pancreatic duct obstruction, secondary to clot formation [1], [5], [6], [7]. Pain is alleviated as the clot leaves the gastrointestinal tract causing melaena, hematemesis, and, occasionally, hematochezia [2], [5], [7]. Elevated serum amylase is related to increased intraductal pressure [5], [6], [7] and to pain itself. On physical examination, less prevalent symptoms include nausea, vomiting, jaundice, and a pulsatile palpable mass [2], [3], [5].

The diagnosis of hemosuccus pancreaticus is often delayed due to its low prevalence [2], [8]. HP is an entity diagnosed based on the clinical course, endoscopic findings, and radiological evidence, but a definitive diagnosis can only be confirmed by angiography [2], [5], [7]. Initial studies in patients presenting with gastrointestinal bleeding are endoscopy and colonoscopy. However, they may not demonstrate active bleeding or the source of the bleeding [3], [4], [5], not ruling out the diagnosis [1], [3]. Contrast computed tomography (CT) or magnetic resonance imaging (MRI) are useful to visualize the presence of pancreatic pathology, its extension, and the level of involvement of the peripancreatic vessels, besides helping to detect tumors, pseudoaneurysms, pseudocysts, or surrounding thrombosis [3], [4], [6], but arteriography of the gastroduodenal, splenic, hepatic, and pancreaticoduodenal arteries is the most specific diagnostic method [5], [6], [7], [8], [9].

There are mainly 2 therapeutic options: interventional radiological procedures and surgery [2], [3], [4], [5]. Surgical intervention is indicated for bleeding from large diameter vessels [2], [3], [5], especially in cases with unsuccessful embolization, uncontrolled bleeding, and persistent hemodynamic compromise [2], [5], [9].

## 2 Presentation of the case

After ethical and institutional approval, previous informed consent filled, and following SCARE guidelines [10]; our paper presents a 70-years-old male patient who presented to the emergency room (ER) with abdominal pain at mesogastrium, associated with melaena and weight loss. Abdominal pain started two months before the emergency room consultation with no associated triggers. There was no prior history of liver disease or gastrointestinal bleeding.

A physical exam at the ER revealed normal arterial pressure (117/78 mm Hg). The patient was pale, diaphoretic, and somnolent, with tachycardia, with abdominal tenderness in mesogastrium radiated to the back, with no peritoneal irritation. Initial laboratory studies revealed metabolic alkalemia with hyperlactatemia up to 2.01 mmol/L, hemoglobin of 13,1 g/dL with normal liver function test. There was no coagulopathy.

**Fig. 1:**
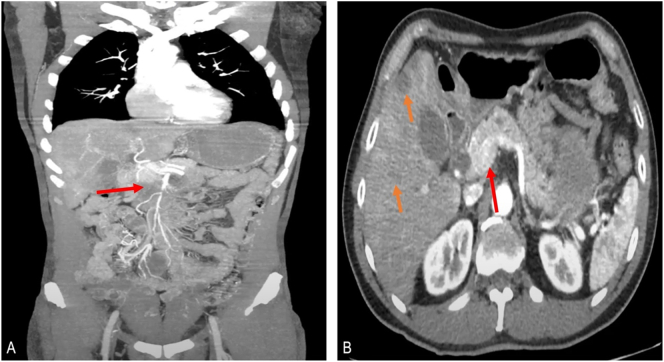
A. Abdominal angiotomography coronal view (red arrow pointing to the pancreatic head mass). B Abdominal angiotomography sagittal view (red arrows pointing to the pancreatic head mass/orange arrows pointing liver metastases).

Due to the clinical course, the initial suspicion was mesenteric ischemia and an emergency abdominal angiotomography was performed. A 38 × 36 mm mass adjacent to the uncinate process of the pancreas with infiltration of the superior mesenteric vein and dilatation of the biliary tract was discovered (Fig. 1).

Esophagogastroduodenoscopy (EGDE) and colonoscopy were performed to further evaluate the cause of gastrointestinal bleeding. Colonoscopy ruled out lower gastrointestinal bleeding. EGDE shows bleeding through the duodenal papilla with a Forrest III ulcer, suggestive of malignancy with stigmata of bleeding of unidentifiable origin. The bile tract and pancreatic duct couldn't be canalized during the procedure.

During hospitalization due to persistent bleeding, the patient presented signs, symptoms, and para-clinical findings of hemodynamic deterioration given by hemoglobin levels of 6.5 g/dL, requiring multiple transfusions of red blood cells and intensive care unit observation. These findings and clinical course required hepatobiliary and pancreatic (HPB) surgery consultation.

In the initial evaluation by the HPB surgery group, amylase levels were requested, with evidence of pronounced hyperamylasemia of 400 U/L completing the Sandblom triad, characteristic of HP syndrome.

**Fig. 2:**
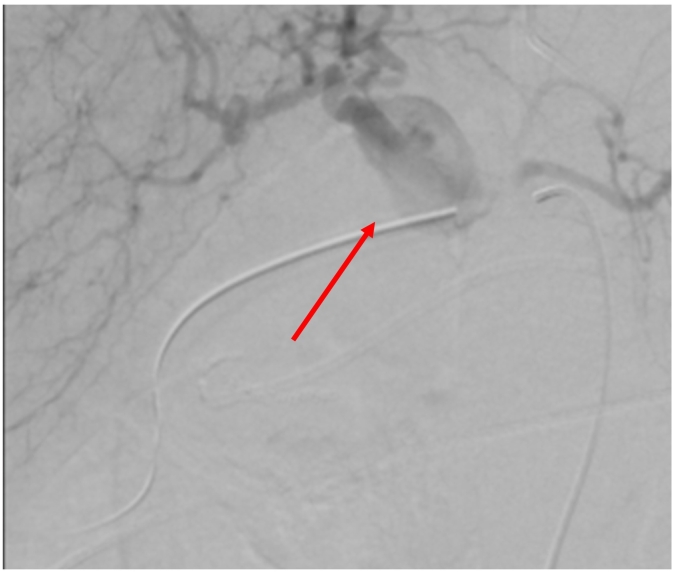
Endovascular view of gastroduodenal artery pseudoaneurysm (red arrow pointing to the pseudoaneurysm).

**Fig. 3:**
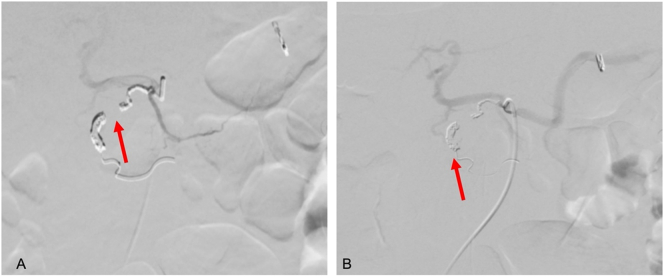
A. Embolization of gastroduodenal artery pseudoaneurysm (red arrow pointing vessel tract). B. Confirmation of coil positioning (red arrow pointing coil).

Due to the persistence of melaena and rectal bleeding associated with new upper gastrointestinal bleeding, a new EGDE was requested with evidence of active layered bleeding in gastric ulcers. Hemostasis was performed with hemoclip. Selective arteriography was performed, where an abrupt interruption of the gastroduodenal artery was found in relation to the pancreatic mass confirming a pseudoaneurysm (Fig. 2). As well, initial suspicion of rupture of the pseudoaneurysm into the duodenum was ruled out according to the arteriography, and for that reason the preferred management was an endovascular coil embolization, which was performed, achieving distal occlusion of the vessel, with no evidence of persisting bleeding on inspection (Fig. 3).

Despite the established management, gastrointestinal bleeding and abdominal pain continue, so an abdominal MRI was requested, which revealed a previously known periampullary tumor dependent on the uncinate process of the pancreas measuring 47 × 42 mm with unresectable characteristics with liver metastases involving all the parenchyma. Third EDGE was requested on suspicion of active bleeding; however, it was ruled out.

In the hematological controls, the hemoglobin level despite red blood cell transfusions was not adequate, and the patient presented signs of hemodynamic compromise, so another angiography was performed, which evidenced a small pseudoaneurysm of the remnant of the gastroduodenal artery, which had been previously embolized. Due to the findings, selective catheterization with Coil endovascular embolization was performed, preserving the hypertrophic common hepatic and pancreatic arteries and selective catheterization of gastric vessels.

After endovascular management for HP syndrome, gastrointestinal bleeding was controlled with no evidence of hemorrhage or hemoglobin level decrease after 96 h of embolization. The patient's clinical condition worsened in relation to his neoplastic pathology and liver metastatic involvement, in the follow-up period after 25 days of management, the patient died due to liver failure and pulmonary embolism.

## 3 Discussion and conclusions

Hemosuccus pancreaticus remains to be an unknown pathology due to its reduced rate of occurrence [3], [4]. In general terms has been estimated to occur in about one in 1500 cases of gastrointestinal bleeding [1], [3], [7]. It is a rare cause of intermittent upper gastrointestinal bleeding [1], [2], [3], [4], [5], more frequently occurs in the male population, and is associated with alcohol consumption as well as the development of acute and chronic pancreatitis in 75–90 % of the cases [1], [3], vascular malformation, pancreatic tumors, pancreas divisum or trauma [2], [6], [7], [9]. The vessels most related to the bleeding include the splenic (40 %), gastroduodenal (30 %), pancreaticoduodenal (20 %), gastric (5 %), and hepatic arteries (2 %) [3], [7]. Nevertheless, in the whole spectrum of etiologies of upper gastrointestinal bleeding, HP due to gastroduodenal artery pseudoaneurysm remains to be an extremely rare clinical entity [2], [4], [9].

The pathogenesis of this phenomenon is related to inflammation due to acute or chronic pancreatitis, which triggers the release of pancreatic enzymes that cause arterial wall injury due to their lytic properties [1], [3], [9]. This endothelial damage enhances the cyst formation and increases the probability of vessel wall rupture and formation of a pseudoaneurysm, leading to decreased resistance and increased blood flow [1], potentially resulting in rupture of the pseudoaneurysm in the pancreatic duct, causing hemorrhage through the major duodenal papilla [1], [3].

The disease is often difficult to diagnose at an early stage due to its rarity, anatomical location, and bleeding patterns that keep it non-visible by endoscopic or radiological methods [5]. The delay in the precise identification of this etiology culminates in delayed management, potentially resulting in high morbidity and mortality [1], [3], [9]. Clinical aspects described are secondary to the increased intraductal pressure due to distention of the pancreatic duct [3], [5], [9] which results in elevated amylase levels [4], intermittent abdominal pain that resolves with the clot clearance, and gastrointestinal bleeding. [6], [8]. The first cases reported in the literature date back to 1970, Sandbloom et al. [6], described the first three cases, explaining the clinical conditions of each patient, and describing a triad that represents the clinical findings in these patients, with epigastric abdominal pain, gastrointestinal bleeding or hemobilia and elevated amylase levels, same findings that the ones evidenced in our cases. This triad is also present in most of the cases reported in the literature [2], [4], [7].

Diagnosis requires a multidisciplinary and integrative approach [1], [2], it may require a combination of endoscopic and imaging modalities. [2], [3], [5]. The use of endoscopic methods remains to be a matter of debate considering that only 30 % [5], [8] of the case's evidence bleeding, therefore angiography and clinical history constitute the key elements of the diagnosis [1].

Computed tomography has been widely described as a feasible diagnostic tool with increased rates of accuracy and helps the surgeon to localize the origin of the bleeding to define a definitive treatment. [12] Multiple CT modalities have been proposed among dynamic CT scans, multidetector enterography, multiphasic CT scans, and dual-energy CT evaluations [12]. All these modalities are rapid and non-invasive techniques with shorter acquisition times that allows a quick study in an emergency context [12]. A dynamic CT scan should be performed as the first study after the endoscopic evaluation in order to localize the origin of the bleeding and to prepare an endovascular or surgical approach to the condition. Nevertheless, CT angiography is recommended when there is a suspicion of active bleeding of upper gastrointestinal or colorectal source and has demonstrated a sensitivity of 96 % in patients with a diagnosis of HP [1], [3]. It identifies the source artery and facilitates the identification of the arterial anatomy for therapeutic intervention in some cases [3]. In fact, tomographic and angiographic images could rule out a vascular emergency such as the intraluminal rupture of the vessel as described by Jiang et al. in his case report [11], which is a rare and life-threatening pathology, in our case due to the suspicion of this emergency, a diagnostic and therapeutic approach was preferred (angiography), and the rupture of the vessel in our case gastroduodenal artery was ruled out.

Management of HP requires the complete eradication of the source of the bleeding [2]. There are two possible methods: interventional radiological procedures and surgery [5], [7], [8]. If the source of bleeding is detected by angiography, interventional radiology procedures such as embolization, balloon tamponade, and stent placement are the first line of choice for initial treatment if the hemodynamic situation is controlled [1], [3], [5], with successful results in >65 % [7] of cases, and rebleeding described in 20 % [7], [9]. The most frequently described technique is endovascular embolization. It promotes thrombus formation in the pseudoaneurysm, occluding the artery. Nevertheless, if the collateral supply isn't enough, it can cause ischemia [7].

Surgical treatment is indicated in uncontrolled hemorrhage, shock, failure of embolization, rebleeding after interventional radiographic approaches, or when the initial angiography shows no abnormal findings [2], [8]. Also is indicated in patients with underlying pathologies such as pancreatic pseudocyst, gastric outlet obstruction, abscess, or resectable periampullary tumor [5]. Literature has shown success rates of surgical management up to 80 % and mortality rates of 23 % to 50 % [4], [7].

Surgical procedures described include intracystic, pseudoaneurysm exclusion with arterial-end ligation, pancreatectomy, pancreaticoduodenectomy, and pancreatic pseudocyst drainage [5], however, arterial-end ligation is not recommended when the bleeding comes from gastroduodenal and pancreaticoduodenal vessels due to the risk of intermittent bleeding, that has been reported up to 5 % [1], [3], [5].

Delayed diagnosis and treatment of HP lead to major complications such as massive gastrointestinal bleeding, chronic anemia, visceral rupture, hemoperitoneum, hemodynamic compromise, multiple organ dysfunction, and death [1], [5], [9]. The mortality associated with this syndrome is reported in 9.6 % [7] of the cases, however, it can increase up to >80 % [7], [8] according to the patient's hemodynamic compromise, the availability of treatment options, and underlying clinical course such as oncologic or traumatic conditions [1], [5]. In our case, endovascular embolization was successful to stop the bleeding, however advanced oncologic diagnosis of the patient leads to an unfortunate outcome for the patient at the follow-up.

According to Ru et al. [3] in the most recent systematic review, the majority of cases report corresponds to Asian, European, and North American population [2], [3], [4], [6], [7], [8], [9]; to the best of our knowledge, this is the first report of hemosuccus pancreaticus in Colombia and in Latin-America.

In conclusion HP is a rare and complex entity; diagnosis and treatment are still challenging. Clinical presentation is related to gastrointestinal bleeding and intermittent abdominal pain with evidence of high amylase levels. Although the diagnosis is challenging, appropriate studies can reduce mortality. The absence of bleeding in the initial endoscopic studies should not dismiss the diagnosis. The multidisciplinary approach, early interventional radiology, and surgical procedures are the cornerstone of management in reducing rates of morbimortality.

## List of abbreviations

HP: Hemosuccus pancreaticus
CT: Contrast computed tomography
MRI: Magnetic resonance imaging
SCARE: Surgical Case Report Guidelines
ER: Emergency room
EGDE: Esophagogastroduodenoscopy
HPB: Hepatobiliary and pancreatic surgery

## Consent for publication

Informed consent of the patient was obtained and is available from the corresponding author on a reasonable request.

## Availability of data and materials

The datasets used and/or analyzed during the current study are available from the corresponding author on reasonable request.

## Ethical approval

Ethical committee approval (Universidad el Rosario) and informed consent of the patient was obtained.

## Funding

The present manuscript does not receive any funding.

## Guarantor

Carlos Rey.

## Research registration number

Do not apply.

## CRediT authorship contribution statement

DA: Manuscript writing, critical revision of the manuscript, data analysis.
FP: Data analysis, manuscript writing.
JG: Data analysis, manuscript writing.
CR: Manuscript writing, critical revision of the manuscript, data analysis.
DC: Manuscript writing, critical revision of the manuscript, data analysis.
JS: Manuscript writing, critical revision of the manuscript, data analysis.

## Declaration of competing interest

The authors declare that they have no competing interest.
